# *N*-(2-Oxo-2*H*-chromen-3-yl)benzamide

**DOI:** 10.1107/S1600536810008275

**Published:** 2010-03-06

**Authors:** Mukesh M. Jotani, Bharat B. Baldaniya, Edward R. T. Tiekink

**Affiliations:** a Department of Physics, Bhavan’s Sheth R. A. College of Science, Ahmedabad, Gujarat 380 001, India; b Department of Chemistry, M. G. Science Institute, Navrangpura, Ahmedabad, Gujarat 380 009, India; c Department of Chemistry, University of Malaya, 50603 Kuala Lumpur, Malaysia

## Abstract

The phenyl ring in title mol­ecule, C_16_H_11_NO_3_, forms a dihedral angle of 7.69 (6)° with the fused ring system. The observed conformation is stabilized by intra­molecular N—H⋯O and C—H⋯O inter­actions. In the crystal, supra­molecular chains are formed along the *b* axis which are mediated by π–π inter­actions [centroid–centroid distance = 3.614 (2) Å].

## Related literature

For the biological activity of imidazoles, see: Yohjiro *et al.* (1990 ▶). For the anti-inflammatory activity of the title compound, see: Maddi *et al.* (2007 ▶). Semi-empirical quantum chemical calculations were performed using *MOPAC2009* Stewart (2009 ▶).
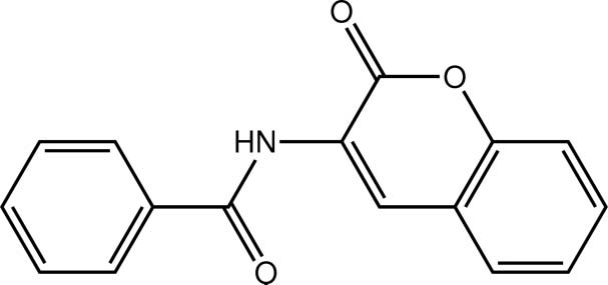


## Experimental

### 

#### Crystal data

C_16_H_11_NO_3_
*M* *_r_* = 265.26
Monoclinic, 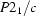

*a* = 12.519 (4) Å
*b* = 4.748 (3) Å
*c* = 21.167 (4) Å
β = 102.044 (3)°
*V* = 1230.5 (9) Å^3^
*Z* = 4
Mo *K*α radiation
μ = 0.10 mm^−1^
*T* = 293 K
0.40 × 0.22 × 0.15 mm

#### Data collection

Bruker SMART APEX CCD diffractometer
Absorption correction: multi-scan (*SADABS*; Sheldrick, 1996 ▶) *T*_min_ = 0.945, *T*_max_ = 0.995
13295 measured reflections
2827 independent reflections
2029 reflections with *I* > 2σ(*I*)
*R*_int_ = 0.025

#### Refinement

*R*[*F* ^2^ > 2σ(*F* ^2^)] = 0.040
*wR*(*F* ^2^) = 0.118
*S* = 1.11
2827 reflections
184 parameters
1 restraint
H atoms treated by a mixture of independent and constrained refinement
Δρ_max_ = 0.18 e Å^−3^
Δρ_min_ = −0.18 e Å^−3^

### 

Data collection: *APEX2* (Bruker, 2004 ▶); cell refinement: *APEX2* and *SAINT* (Bruker, 2004 ▶); data reduction: *SAINT* and *XPREP* (Bruker, 2004 ▶); program(s) used to solve structure: *SHELXS97* (Sheldrick, 2008 ▶); program(s) used to refine structure: *SHELXL97* (Sheldrick, 2008 ▶); molecular graphics: *ORTEP-3* (Farrugia, 1997 ▶) and *DIAMOND* (Brandenburg, 2006 ▶); software used to prepare material for publication: *publCIF* (Westrip, 2010 ▶).

## Supplementary Material

Crystal structure: contains datablocks global, I. DOI: 10.1107/S1600536810008275/hg2654sup1.cif

Structure factors: contains datablocks I. DOI: 10.1107/S1600536810008275/hg2654Isup2.hkl

Additional supplementary materials:  crystallographic information; 3D view; checkCIF report

## Figures and Tables

**Table 1:** Hydrogen-bond geometry (Å, °)

*D*—H⋯*A*	*D*—H	H⋯*A*	*D*⋯*A*	*D*—H⋯*A*
N1—H1n⋯O1	0.87 (1)	2.24 (2)	2.659 (2)	110 (1)
C8—H8⋯O3	0.93	2.24	2.822 (3)	120

**Fig. 1.**
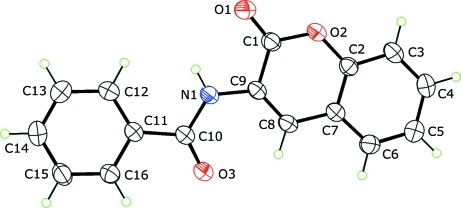
The molecular structure of (I) showing the atom-labelling scheme and displacement ellipsoids at the 35% probability level.

**Fig. 2.**
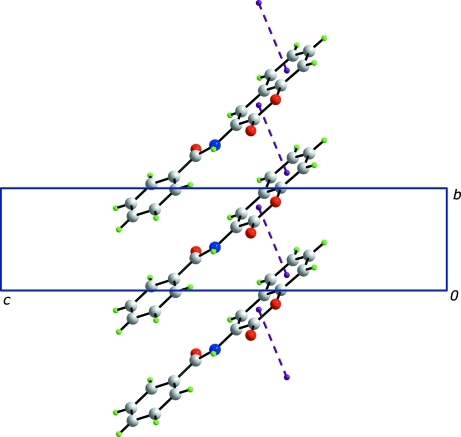
A supramolecular chain aligned along the b axis in (I), mediated by π–π interactions (purple dashed lines). Colour code: O, red; N, blue; C, grey; and H, green.

## supplementary crystallographic information

### Comment

Oxazoles are very useful synthetic intermediates used for the generation of imidazoles that possess a wide spectrum of biological activities such as herbicidal, anti-bacterial, anti-fungal, *etc*. (Yohjiro *et al.*, 1990). During attempts designed to synthesize oxazoles containing various substituents at different positions in the benzene ring, the title compound, (I), was obtained unexpectedly by the formation of a chromen derivative instead of the anticipated oxazole. Compound (I) is a known species and has been shown to be pharmaceutically important as it possesses anti-inflammatory activity (Maddi *et al.*, 2007).

There is a twist in the molecule of (I), Fig. 1, so that the pendent benzene ring is not co-planar with the rest of the molecule. This is seen in the value of the O3–C10–C11–C12 torsion angle of -166.43 (15) °, and in the dihedral formed between the fused ring system and benzene ring of 7.69 (6) °. The overall conformation of the molecule is stabilised by intramolecular N–H···O and C–H···O interactions, Table 1, which close S(5) and S(6) hydrogen bond ring motifs, respectively. The most prominent feature of the crystal packing is the formation of supramolecular chains along the *b* axis mediated by π–π interactions between the ring centroids of the (O2,C1,C2,C7—C9) and (C2—C7)^i^ rings of 3.614 (2) Å of translationally related molecules; symmetry operation *i*: *x*, 1+*y*, *z*.

Semi-empirical Quantum Chemical Calculations were performed on experimental structure using MOPAC2009 program (Stewart, 2009) to optimize the molecule with the Austin Model 1 (AM1) approximation, together with the restricted Hartree-Fock closed-shell wavefunction. Minimisations were terminated at an r.m.s. gradient of less than 1.0 kJ mol^-1^ Å^-1^. These calculations gave a heat of formation = -213.437 kJ for (I). The ionization potential, dipole moment and self consistency field (SCF) factor were calculated as 9.033 eV, 1.798 Debye and 73, respectively.

### Experimental

A mixture of orthohydrothoxy benzaldehyde (0.25 mol), benzoyl amino acetic acid (0.25 mol), acetyl acetate (0.30 mol), and anhydrous sodium acetate (0.25 mol) were taken in a 500 ml round bottom flask and heated on an electric hot plate with constant stirring. After complete liquefaction, the flask was transferred to a sand bath and further heated for 2 h. Ethanol (100 ml) was added slowly to the flask and the mixture was allowed to stand overnight. The crystalline product obtained was filtered with ice-cold alcohol and then with boiling water. The crude product was crystallised from ethanol (95%) to obtain the final product (75 % yield, m.pt. 432 K). The colorless crystals were obtained by slow evaporation from an ethanol solution.

### Refinement

The C-bound H atoms were geometrically placed (C–H = 0.93 Å) and refined as riding with *U_iso_*(H) = 1.2*U_eq_*(parent atom). The position of the N–H atom was refined with *U_iso_*(H) = 1.2*U_eq_*(N).

### Crystal data

**Table:**

C_16_H_11_NO_3_	*F*(000) = 552
*M**_r_* = 265.26	*D*_x_ = 1.432 Mg m^−^^3^
Monoclinic, *P*2_1_/*c*	Mo *K*α radiation, λ = 0.71073 Å
Hall symbol: -P 2ybc	Cell parameters from 3699 reflections
*a* = 12.519 (4) Å	θ = 2.3–29.6°
*b* = 4.748 (3) Å	µ = 0.10 mm^−^^1^
*c* = 21.167 (4) Å	*T* = 293 K
β = 102.044 (3)°	Block, colourless
*V* = 1230.5 (9) Å^3^	0.40 × 0.22 × 0.15 mm
*Z* = 4	

### Data collection

**Table:**

Bruker SMART APEX CCD diffractometer	2827 independent reflections
Radiation source: fine-focus sealed tube	2029 reflections with *I* > 2σ(*I*)
graphite	*R*_int_ = 0.025
ω and φ scans	θ_max_ = 27.5°, θ_min_ = 1.7°
Absorption correction: multi-scan (*SADABS*; Sheldrick, 1996)	*h* = −16→15
*T*_min_ = 0.945, *T*_max_ = 0.995	*k* = −6→5
13295 measured reflections	*l* = −27→27

### Refinement

**Table:**

Refinement on *F*^2^	Primary atom site location: structure-invariant direct methods
Least-squares matrix: full	Secondary atom site location: difference Fourier map
*R*[*F*^2^ > 2σ(*F*^2^)] = 0.040	Hydrogen site location: inferred from neighbouring sites
*wR*(*F*^2^) = 0.118	H atoms treated by a mixture of independent and constrained refinement
*S* = 1.11	*w* = 1/[σ^2^(*F*_o_^2^) + (0.0491*P*)^2^ + 0.2124*P*] where *P* = (*F*_o_^2^ + 2*F*_c_^2^)/3
2827 reflections	(Δ/σ)_max_ = 0.001
184 parameters	Δρ_max_ = 0.18 e Å^−^^3^
1 restraint	Δρ_min_ = −0.18 e Å^−^^3^

### Special details

**Table:**

Geometry. All s.u.'s (except the s.u. in the dihedral angle between two l.s. planes) are estimated using the full covariance matrix. The cell s.u.'s are taken into account individually in the estimation of s.u.'s in distances, angles and torsion angles; correlations between s.u.'s in cell parameters are only used when they are defined by crystal symmetry. An approximate (isotropic) treatment of cell s.u.'s is used for estimating s.u.'s involving l.s. planes.
Refinement. Refinement of *F*^2^ against ALL reflections. The weighted *R*-factor wR and goodness of fit *S* are based on *F*^2^, conventional *R*-factors *R* are based on *F*, with *F* set to zero for negative *F*^2^. The threshold expression of *F*^2^ > 2σ(*F*^2^) is used only for calculating *R*-factors(gt) etc. and is not relevant to the choice of reflections for refinement. *R*-factors based on *F*^2^ are statistically about twice as large as those based on *F*, and *R*- factors based on ALL data will be even larger.

### Fractional atomic coordinates and isotropic or equivalent isotropic displacement parameters (Å^2^)

**Table:**

	*x*	*y*	*z*	*U*_iso_*/*U*_eq_	
O1	0.57658 (10)	0.4385 (3)	0.56046 (6)	0.0705 (4)	
O2	0.69047 (9)	0.1338 (2)	0.61603 (5)	0.0526 (3)	
O3	0.85472 (10)	0.6200 (3)	0.43753 (6)	0.0667 (4)	
N1	0.70508 (10)	0.5787 (3)	0.48003 (6)	0.0443 (3)	
H1N	0.6366 (8)	0.619 (4)	0.4767 (8)	0.053*	
C1	0.66406 (13)	0.3252 (3)	0.56770 (7)	0.0469 (4)	
C2	0.78915 (12)	−0.0059 (3)	0.62787 (7)	0.0426 (3)	
C3	0.80701 (14)	−0.1960 (3)	0.67819 (7)	0.0534 (4)	
H3	0.7545	−0.2255	0.7027	0.064*	
C4	0.90379 (15)	−0.3405 (4)	0.69126 (8)	0.0555 (4)	
H4	0.9174	−0.4688	0.7252	0.067*	
C5	0.98102 (14)	−0.2972 (4)	0.65453 (8)	0.0552 (4)	
H5	1.0463	−0.3974	0.6635	0.066*	
C6	0.96208 (13)	−0.1070 (3)	0.60475 (8)	0.0509 (4)	
H6	1.0148	−0.0793	0.5802	0.061*	
C7	0.86511 (12)	0.0452 (3)	0.59038 (7)	0.0414 (3)	
C8	0.84018 (12)	0.2473 (3)	0.53927 (7)	0.0440 (4)	
H8	0.8909	0.2863	0.5140	0.053*	
C9	0.74390 (12)	0.3800 (3)	0.52784 (6)	0.0403 (3)	
C10	0.76069 (13)	0.6840 (3)	0.43656 (7)	0.0440 (4)	
C11	0.70075 (12)	0.8878 (3)	0.38827 (7)	0.0408 (3)	
C12	0.60296 (13)	1.0138 (3)	0.39149 (7)	0.0500 (4)	
H12	0.5684	0.9685	0.4250	0.060*	
C13	0.55600 (14)	1.2065 (4)	0.34538 (8)	0.0579 (4)	
H13	0.4901	1.2911	0.3481	0.069*	
C14	0.60574 (15)	1.2743 (4)	0.29565 (8)	0.0584 (5)	
H14	0.5738	1.4047	0.2646	0.070*	
C15	0.70244 (15)	1.1497 (4)	0.29178 (8)	0.0590 (5)	
H15	0.7360	1.1945	0.2578	0.071*	
C16	0.75030 (14)	0.9590 (3)	0.33763 (7)	0.0510 (4)	
H16	0.8165	0.8765	0.3348	0.061*	

### Atomic displacement parameters (Å^2^)

**Table:**

	*U*^11^	*U*^22^	*U*^33^	*U*^12^	*U*^13^	*U*^23^
O1	0.0528 (7)	0.0950 (10)	0.0687 (8)	0.0157 (7)	0.0238 (6)	0.0260 (7)
O2	0.0523 (7)	0.0612 (7)	0.0488 (6)	0.0031 (5)	0.0209 (5)	0.0136 (5)
O3	0.0563 (7)	0.0819 (9)	0.0682 (8)	0.0137 (6)	0.0275 (6)	0.0313 (7)
N1	0.0458 (7)	0.0477 (7)	0.0413 (7)	−0.0018 (6)	0.0135 (6)	0.0049 (5)
C1	0.0461 (9)	0.0543 (9)	0.0415 (8)	−0.0016 (7)	0.0118 (6)	0.0042 (7)
C2	0.0472 (8)	0.0423 (8)	0.0390 (7)	−0.0038 (6)	0.0106 (6)	−0.0016 (6)
C3	0.0634 (10)	0.0557 (9)	0.0434 (8)	−0.0038 (8)	0.0167 (7)	0.0066 (7)
C4	0.0685 (11)	0.0486 (9)	0.0471 (9)	−0.0004 (8)	0.0063 (8)	0.0074 (7)
C5	0.0551 (10)	0.0509 (9)	0.0572 (10)	0.0028 (8)	0.0064 (8)	0.0012 (8)
C6	0.0508 (9)	0.0525 (9)	0.0512 (9)	−0.0005 (7)	0.0149 (7)	−0.0009 (7)
C7	0.0491 (8)	0.0388 (7)	0.0373 (7)	−0.0063 (6)	0.0112 (6)	−0.0043 (6)
C8	0.0504 (9)	0.0449 (8)	0.0401 (7)	−0.0046 (7)	0.0175 (6)	−0.0009 (6)
C9	0.0478 (8)	0.0399 (7)	0.0344 (7)	−0.0063 (6)	0.0115 (6)	−0.0014 (6)
C10	0.0487 (9)	0.0443 (8)	0.0407 (8)	−0.0043 (7)	0.0130 (6)	0.0005 (6)
C11	0.0474 (8)	0.0375 (7)	0.0376 (7)	−0.0066 (6)	0.0090 (6)	−0.0024 (6)
C12	0.0514 (9)	0.0547 (9)	0.0462 (8)	−0.0042 (8)	0.0157 (7)	0.0005 (7)
C13	0.0512 (10)	0.0578 (10)	0.0628 (10)	0.0063 (8)	0.0075 (8)	−0.0003 (8)
C14	0.0706 (12)	0.0501 (9)	0.0506 (9)	0.0028 (8)	0.0037 (8)	0.0078 (8)
C15	0.0740 (12)	0.0572 (10)	0.0485 (9)	0.0024 (9)	0.0191 (8)	0.0113 (8)
C16	0.0570 (9)	0.0527 (9)	0.0463 (8)	0.0035 (8)	0.0177 (7)	0.0036 (7)

### Geometric parameters (Å, °)

**Table:**

O1—C1	1.2009 (19)	C6—H6	0.9300
O2—C1	1.3567 (18)	C7—C8	1.431 (2)
O2—C2	1.3783 (18)	C8—C9	1.336 (2)
O3—C10	1.2118 (18)	C8—H8	0.9300
N1—C10	1.3596 (19)	C10—C11	1.492 (2)
N1—C9	1.3947 (19)	C11—C12	1.377 (2)
N1—H1N	0.867 (9)	C11—C16	1.388 (2)
C1—C9	1.460 (2)	C12—C13	1.377 (2)
C2—C3	1.378 (2)	C12—H12	0.9300
C2—C7	1.381 (2)	C13—C14	1.369 (3)
C3—C4	1.369 (2)	C13—H13	0.9300
C3—H3	0.9300	C14—C15	1.365 (2)
C4—C5	1.377 (2)	C14—H14	0.9300
C4—H4	0.9300	C15—C16	1.371 (2)
C5—C6	1.370 (2)	C15—H15	0.9300
C5—H5	0.9300	C16—H16	0.9300
C6—C7	1.391 (2)		
			
C1—O2—C2	121.85 (12)	C9—C8—H8	120.0
C10—N1—C9	126.13 (13)	C7—C8—H8	120.0
C10—N1—H1N	120.0 (11)	C8—C9—N1	127.93 (14)
C9—N1—H1N	113.5 (11)	C8—C9—C1	120.79 (13)
O1—C1—O2	117.93 (14)	N1—C9—C1	111.28 (13)
O1—C1—C9	124.25 (14)	O3—C10—N1	121.95 (14)
O2—C1—C9	117.81 (14)	O3—C10—C11	121.49 (13)
O2—C2—C3	116.77 (14)	N1—C10—C11	116.55 (14)
O2—C2—C7	120.63 (13)	C12—C11—C16	118.54 (14)
C3—C2—C7	122.60 (15)	C12—C11—C10	124.99 (14)
C4—C3—C2	118.57 (16)	C16—C11—C10	116.44 (14)
C4—C3—H3	120.7	C11—C12—C13	120.41 (15)
C2—C3—H3	120.7	C11—C12—H12	119.8
C3—C4—C5	120.47 (15)	C13—C12—H12	119.8
C3—C4—H4	119.8	C14—C13—C12	120.40 (17)
C5—C4—H4	119.8	C14—C13—H13	119.8
C6—C5—C4	120.23 (16)	C12—C13—H13	119.8
C6—C5—H5	119.9	C15—C14—C13	119.71 (16)
C4—C5—H5	119.9	C15—C14—H14	120.1
C5—C6—C7	120.90 (15)	C13—C14—H14	120.1
C5—C6—H6	119.5	C14—C15—C16	120.40 (16)
C7—C6—H6	119.5	C14—C15—H15	119.8
C2—C7—C6	117.21 (14)	C16—C15—H15	119.8
C2—C7—C8	118.97 (14)	C15—C16—C11	120.53 (16)
C6—C7—C8	123.82 (14)	C15—C16—H16	119.7
C9—C8—C7	119.94 (14)	C11—C16—H16	119.7
			
C2—O2—C1—O1	−179.90 (14)	C10—N1—C9—C1	177.37 (14)
C2—O2—C1—C9	0.1 (2)	O1—C1—C9—C8	179.34 (16)
C1—O2—C2—C3	−179.91 (13)	O2—C1—C9—C8	−0.7 (2)
C1—O2—C2—C7	0.0 (2)	O1—C1—C9—N1	−0.5 (2)
O2—C2—C3—C4	179.59 (14)	O2—C1—C9—N1	179.48 (12)
C7—C2—C3—C4	−0.3 (2)	C9—N1—C10—O3	−3.4 (2)
C2—C3—C4—C5	−0.4 (2)	C9—N1—C10—C11	177.89 (13)
C3—C4—C5—C6	0.5 (3)	O3—C10—C11—C12	−166.43 (15)
C4—C5—C6—C7	0.0 (2)	N1—C10—C11—C12	12.3 (2)
O2—C2—C7—C6	−179.11 (13)	O3—C10—C11—C16	11.7 (2)
C3—C2—C7—C6	0.7 (2)	N1—C10—C11—C16	−169.62 (14)
O2—C2—C7—C8	0.5 (2)	C16—C11—C12—C13	−0.2 (2)
C3—C2—C7—C8	−179.65 (14)	C10—C11—C12—C13	177.90 (14)
C5—C6—C7—C2	−0.6 (2)	C11—C12—C13—C14	0.3 (3)
C5—C6—C7—C8	179.82 (14)	C12—C13—C14—C15	0.1 (3)
C2—C7—C8—C9	−1.0 (2)	C13—C14—C15—C16	−0.5 (3)
C6—C7—C8—C9	178.53 (14)	C14—C15—C16—C11	0.6 (3)
C7—C8—C9—N1	−179.05 (13)	C12—C11—C16—C15	−0.3 (2)
C7—C8—C9—C1	1.1 (2)	C10—C11—C16—C15	−178.48 (14)
C10—N1—C9—C8	−2.5 (2)		

### Hydrogen-bond geometry (Å, °)

**Table:**

*D*—H···*A*	*D*—H	H···*A*	*D*···*A*	*D*—H···*A*
N1—H1n···O1	0.867 (11)	2.236 (16)	2.659 (2)	110.0 (14)
C8—H8···O3	0.93	2.24	2.822 (3)	120
